# *In Vivo* FRET Imaging Revealed a Regulatory Role of RanGTP in Kinetochore-Microtubule Attachments via Aurora B Kinase

**DOI:** 10.1371/journal.pone.0045836

**Published:** 2012-09-28

**Authors:** Yoke-Peng Lee, Chi-Hang Wong, Kheng-Sze Chan, Soak-Kuan Lai, Cheng-Gee Koh, Hoi-Yeung Li

**Affiliations:** Division of Molecular and Cell Biology, School of Biological Sciences, College of Science, Nanyang Technological University, Singapore; Virginia Tech, United States of America

## Abstract

Under the fluctuating circumstances provided by the innate dynamics of microtubules and opposing tensions resulted from microtubule-associated motors, it is vital to ensure stable kinetochore-microtubule attachments for accurate segregation. However, a comprehensive understanding of how this regulation is mechanistically achieved remains elusive. Using our newly designed live cell FRET time-lapse imaging, we found that post-metaphase RanGTP is crucial in the maintenance of stable kinetochore-microtubule attachments by regulating Aurora B kinase via the NES-bearing Mst1. More importantly, our study demonstrates that by ensuring stable alignment of metaphase chromosomes prior to segregation, RanGTP is indispensible in governing the genomic integrity and the fidelity of cell cycle progression. Our findings suggest an additional role of RanGTP beyond its known function in mitotic spindle assembly during the prometaphase-metaphase transition.

## Introduction

A key feature in mitosis lies in the preservation of genomic integrity from parent to daughter cells. This is especially critical at metaphase when mitotic chromosomes are aligned along the metaphase plate and are held at the kinetochores by spindle microtubules, in preparation for segregation to daughter cells. Therefore, stable end-on kinetochore-microtubule attachments are essential to attain amphitelic attachments and for mitotic progression through the spindle assembly checkpoint (SAC) with equal partitioning of genetic materials [1]. Early studies on kinetochore-microtubule attachments have been concentrated on the conserved KMN network (KNL1-Mis12-Ndc80/Hec1) in establishing the initial kinetochore-microtubule contact [2]. Impaired Hec1 function in particular, through antibody microinjection or siRNA silencing, results in erroneous attachments, impaired chromosome alignment, and subsequent chromosome mis-segregation [3], [4], [5]. However, with increasing interest on the kinetochore-microtubule network, a plethora of kinetochore and centromeric proteins have been unveiled, many of which with functions yet to be fully determined [6], [7]. Since the kinetochore components constitute the immediate contact between chromosomes and microtubules, the precise regulation of the formation and maintenance of kinetochore-microtubule attachment is thus important to preserve the precision and accuracy of chromosome segregation.

The small GTPase Ran regulates several key events to maintain the fidelity of mitotic chromosome segregation and for proper mitotic progression. The functionalities of Ran hinge on its nucleotide state, whereby the GTP-bound form and GDP-bound form are established by the activities of guanine exchange factor RCC1 and RanGAP1/RanBP1 respectively. During mitosis, there is a high RanGTP level surrounding RCC1-associated chromosomes and progressively decreases towards the cell periphery, generating a RanGTP gradient [8], [9]. This RanGTP gradient dictates Ran’s functions in mitosis. It has been shown that high level of RanGTP surrounds the mitotic chromosomes and sequesters importin alpha/beta, resulting in the release of the NLS (nuclear localization signal)-containing spindle assembly factors from importin for their functions during the prometaphase-metaphase transition [10], [11], [12]. However, little is known about how RanGTP, NES (nuclear export signal)-bearing proteins and exportin Crm1 regulate mitotic progression. We report here, with the application of our newly designed *in vivo* FRET imaging, the post-metaphase function of RanGTP in maintaining stable kinetochore-microtubule attachments after proper chromosome congression by regulating Aurora B kinase via the NES-bearing Mst1.

## Results

### RanGTP is Important for the Maintenance of Proper Chromosome Alignment at the Metaphase Plate

The long speculated existence and functions of the RanGTP gradient during mitosis had since been validated by computational/mathematical models, biosensor visualizations, and the associated *in vitro* and *in vivo* studies [13], [14], [15]. Nonetheless, the mechanistic pathways underlying the regulatory roles of RanGTP remains incompletely elucidated throughout mitosis due to the limitations of the various systems used. To solve this, we first set out to establish a system whereby we can manipulate and monitor the mitotic RanGTP levels in real time by time-lapse imaging. The temperature sensitive tsBN2 cells were chosen for this study. Due to mutation in the RCC1 gene, incubation of tsBN2 cells at non-permissive temperature (39.5°C) leads to depletion of RCC1 protein and RanGTP subsequently [16]. As a measure of the RanGTP level, we utilized the Rango FRET biosensor [8]. To our knowledge, no data depicting the real time depletion of RanGTP level has been reported.

**Figure 1:**
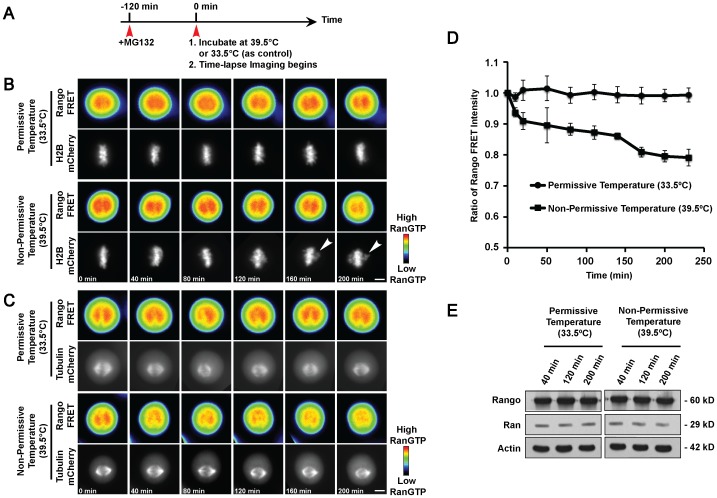
RanGTP is depleted at non-permissive temperature and affects maintenance of chromosome alignment in tsBN2 cells. A) Schematic depiction of the experimental conditions. Cells were arrested at metaphase using MG132 for 2 hours, prior to incubation at permissive (33.5°C) or non-permissive temperature. B) tsBN2 cells expressing Rango and H2B-mCherry. C) tsBN2 cells expressing Rango and tubulin-mCherry. Control experiments at 33.5°C and temperature-shift experiments at 39.5°C. Color bar represents FRET intensity. Scale bar: 10 µm. D) Line chart representation the Rango FRET ratio (according to Youvan’s method) at various time-lapse intervals for control, 33.5°C (round markers), and temperature-shifted, 39.5°C (square markers) cells. Error bars represent ± standard deviation (s.d.). E) Western blot analysis of Rango and Ran from mitotic tsBN2 cells incubated at permissive or non-permissive temperature for various time points. Actin was used as loading control.

Time-lapse imaging using tsBN2 cells co-transfected with either Rango and H2B-mCherry (Fig. 1B) or Rango and tubulin-mCherry (Fig. 1C) were performed. Since the RanGTP was most obvious and accumulated maximally around metaphase chromosomes, the transfected tsBN2 cells were arrested at metaphase using MG132 for 2 hours, prior to incubation at permissive (33.5°C) or non-permissive temperature (Fig. 1A). Consistently, the FRET ratio for both the controls (Fig. 1B and 1C, color encoding images) remained evenly high surrounding the metaphase chromosomes, whereas a decline was measured in cells incubated at non-permissive temperature as the time-lapse progressed (Fig. 1B and 1C, color encoding images). The FRET ratios for cells incubated at both permissive and non-permissive temperature during the time-lapse experiments were plotted as shown in Fig. 1D. This data strongly suggest that the RanGTP level can be manipulated and monitored with our newly designed *in vivo* FRET imaging method.

Interestingly, we found that metaphase-aligned chromosomes progressively moved away from the metaphase plate for tsBN2 cells incubated at non-permissive temperature (Fig. 1B). In contrast, control cells (permissive temperature) exhibit normal metaphase chromosome alignment when RanGTP levels were unperturbed (Fig. 1B). This was unexpected considering that the cells had already achieved stable kinetochore-microtubule attachments for proper chromosome alignment at the metaphase plate prior to incubation at non-permissive temperature. Upon closer inspection, we ruled out the possibility that the change in temperature could compromise mitotic spindle integrity as the cause for this phenotype because the metaphase spindle remains intact following temperature increase (Fig. 1C).

To ascertain that the changes in FRET ratio observed in cells incubated at non-permissive temperature during the time-lapse was not attributed to degradation of the Rango probe or changes in Ran levels upon temperature increase, we probed for these proteins. Rango and Ran levels remained consistent for both control and temperature-shifted mitotic cell lysates. This showed that the Rango probe remained intact following temperature increase and any decline in FRET ratio is attributed to the decrease in RanGTP level.

We next sought to clarify the contribution of the diminished RanGTP as the instigator behind the observed chromosomal misalignment phenotype. Although Rango is widely used for RanGTP visualization purposes, the overabundance of the importin-β binding (IBB) domain potentially generates an “importin-β sink” effect, thereby affecting downstream regulations and functions. Hence, it is necessary to exclude the possibility that the phenotype observed is due to overexpression of Rango.

**Figure 2:**
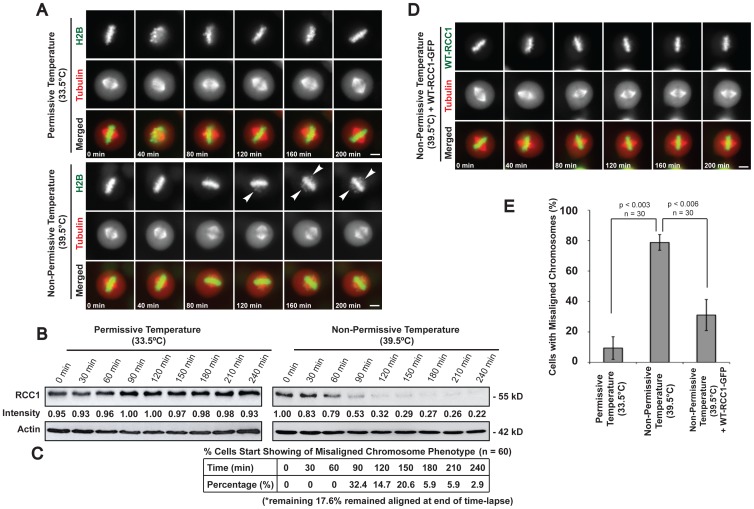
RCC1 degradation is responsible for the chromosome misalignment phenotype at non-permissive temperature. A) Time-lapse imaging of metaphase tsBN2 cells expressing H2B-GFP and tubulin-mCherry. Control experiment at permissive and temperature-shift experiment at non-permissive temperature. Arrows show aberrantly aligned metaphase chromosomes. B) Western blot analysis of RCC1 for mitotic tsBN2 cells incubated at permissive or non-permissive temperature and harvested via mechanical shake-off at various time points. Relative intensity of RCC1 is shown below each lane. Actin was used as loading control. C) Table shows quantification of the proportion of time-lapse imaged cells at the time of appearance of misaligned chromosomes. D) Time-lapse imaging of mitotic tsBN2 cells expressing wild-type RCC1-GFP and tubulin-mCherry. Expression of wild-type RCC1-GFP in metaphase tsBN2 cells incubated at non-permissive temperature abrogated the misalignment phenotype. Scale bar: 10 µm. E) Histogram shows percentage of time-lapse imaged metaphase tsBN2 cells with misaligned chromosomes. Error bars show ± s.d. from three independent experiments. (Student’s t-test).

Therefore, live cell imaging experiments were conducted on tsBN2 cells without Rango transfection. Our results showed that metaphase cells incubated at non-permissive temperature exhibited similar chromosome misalignment, as depicted by the H2B-GFP images of chromosomes displaced from the metaphase plate (Fig. 2A). As the time-lapse progressed, more chromosomes escaped from the metaphase plate. Importantly, although gross chromosome misalignment was observed, the metaphase spindle remained intact and unperturbed by the temperature increase (Fig. 2A, lower panel). Control cells incubated at permissive temperature continued to display properly aligned chromosomes at the metaphase plate till the end of the time-lapse (Fig. 2A, upper panel). As predicted, incubation of cells at non-permissive temperature showed severe decline in RCC1 levels as seen in the immunoblotting analysis of RCC1 in Fig. 2B. Quantification of the proportion of time-lapse imaged metaphase cells at the time of start showing misaligned chromosome phenotype indicates that the degradation of RCC1 and the subsequent depletion of RanGTP precede the occurrence of misaligned chromosomes (Fig. 2C). Importantly, control experiments conducted on the parental BHK21 cell line (with endogenous wild-type RCC1 gene) at non-permissive temperature also showed that temperature increase does not affect the integrity of the mitotic spindle or chromosome alignment at metaphase (Fig. S1). Therefore, attributing the observed chromosome misalignment phenotype in tsBN2 cells at non-permissive temperature solely to the loss of RCC1 and RanGTP depletion.

Further immunoblotting analysis was performed on mitotic cell lysates harvested at various time points after incubation at permissive or non-permissive temperature and probed with antibodies as indicated in Fig. S2A. Protein levels of securin, SMC1 and cdc2 (Thr161) did not show any decline, and chromosomes spread showed intact sister chromatids, indicating that the sister chromatids are attached and that the cells were arrested at metaphase with the anaphase-promoting complex (APC) still inhibited (Fig. S2A–D). Most importantly, Ran regulators, RanGAP1, SUMO-modified RanGAP1 and RanBP1 levels, did not show any significant differences in both control and temperature-shifted cell lysates (Fig. S2A) or in immunofluorescence data (Fig. S2E, F). Therefore, we concluded that the change observed is likely attributed to the loss of RCC1.

To further support that the observed phenotype is a result of depletion of RanGTP during metaphase, we complemented tsBN2 cells with functional wild-type (WT) RCC1 proteins to avert the aberrant chromosomal alignment even when the cells are incubated at non-permissive temperature. We show that 1.5× protein level of the ectopic WT RCC1 relative to the endogenous temperature sensitive RCC1 (data not shown) is necessary and sufficient to rescue the misalignment phenotype at non-permissive temperature (Fig. 2D–E). Thus far, our observations show that the depletion of RanGTP causes an unexpected displacement of chromosomes away from the metaphase plate without compromising the spindle structure at metaphase, suggesting that RanGTP is necessary for the maintenance of proper chromosome alignment during metaphase.

### Local Chromosomal RanGTP Enrichment Regulates the Stability of Kinetochore-microtubule Attachments and Prevents Reactivation of the Spindle Assembly Checkpoint

**Figure 3:**
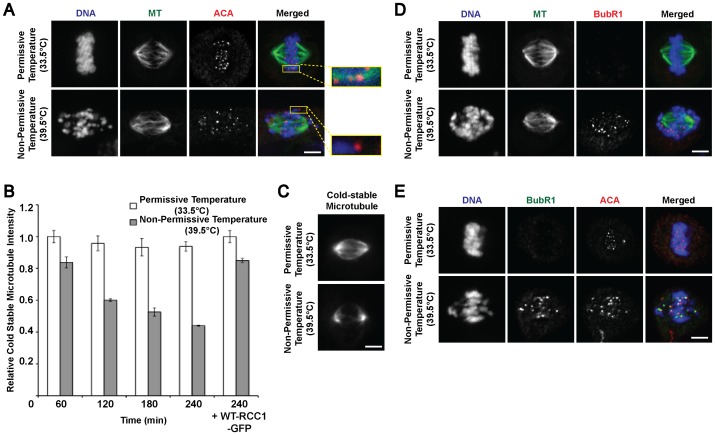
Mitotic RanGTP is required for proper kinetochore-microtubule attachments during metaphase. A) Metaphase tsBN2 Cells were immunostained with anti-centromeric antigens (ACA) and tubulin antibodies. Magnified images show end-on kinetochore-microtubule attachments (control, upper panel) and unattached chromosomes (temperature-shifted, lower panel). B) Histogram shows total cold-stable microtubule intensity (A.U.) after cold-induced microtubule depolymerization. Error bars show ± s.d. (Student’s t-test). C) Representative images of cold-treated mitotic spindles from mitotic tsBN2 cells incubated at permissive or non-permissive temperature. Immunostaining of spindle checkpoint protein BubRI with D) tubulin or E) anti-centromeric ACA. Scale bar: 10 µm.

Aberrant chromosomes displacement from the metaphase plate, as shown in Figure 1 and 2, could be due to improper kinetochore-microtubule attachments or loss of attachment to spindle microtubules [17], [18]. To address these likelihoods, we stained metaphase cells incubated at permissive or non-permissive temperature with anti-centromeric antigens (ACA) and tubulin antibodies. Control cells displayed localization of ACA on chromosomes aligned at the metaphase plate and attached to spindle microtubules (Fig. 3A). For cells incubated at non-permissive temperature, ACA are also detected on the misaligned chromosomes indicating that these chromosomes still have intact centromeres. Magnified images showed proper ACA-microtubule attachments in control cells (Fig. 3A, upper row, second panel) whereas such attachments were compromised in the temperature-shifted cells (Fig. 3A, lower row, second panel).

Stable kinetochore-microtubule attachments confer resistance to microtubule depolymerization during cold exposure whereas unattached microtubules are depolymerized during low temperature treatment [4], [19]. To corroborate our previous data (Fig. 3A), we subjected the cells to cold-treatment for further immunocytochemistry analysis. Quantified relative signal intensities of microtubules revealed that the proportion of cold-stable microtubules was significantly reduced in cells incubated at non-permissive temperature as compared to the control cells (Fig. 3B–C). In agreement, this loss of cold-stable microtubules was averted in metaphase cells expressing wild-type RCC1 (Fig. 3B). These results indicated that the misaligned chromosomes have lost proper end-on kinetochore-microtubule attachments after mitotic RanGTP is depleted during metaphase.

On a separate note, we investigated the localization of several motor or kinetochore proteins, which may be involved in the maintenance or movement of chromosomes during mitosis. Immunostaining results showed that localization of TPX2, Hec1, RanGAP1 and dynactin-p150 were unchanged in metaphase cells incubated in permissive or non-permissive temperature, thus indicating that these proteins are not involved in the manifestation of this phenotype (Fig. S3).

As the SAC remains active until all kinetochore occupancy and kinetochore-spindle tension requirements are satisfied, we reasoned that any unattached kinetochores would then exhibit persisted checkpoint complex localization [20], [21]. Consistent with this, control metaphase cells immunostained with anti-BubR1 showed little or no BubR1 at the kinetochores. In contrast, temperature-shifted cells showed distinctive presence of BubR1 both on the kinetochores of chromosomes displaced from the metaphase plate and to a lesser extent on those that remained at the equator (Fig. 3D). Co-immunostaining with ACA confirmed that the re-assembly of spindle checkpoint apparatus occurs within the vicinity of chromosomal centromeric regions (Fig. 3E). We conclude that the mitotic RanGTP is important in maintaining stable kinetochore-microtubule attachments, and hence restricting any untimely reactivation of SAC.

### RanGTP-dependent Crm1-directed, Mst1-Aurora B Kinase Interactions Maintains Stable Metaphase Kinetochore-microtubule Attachments

**Figure 4:**
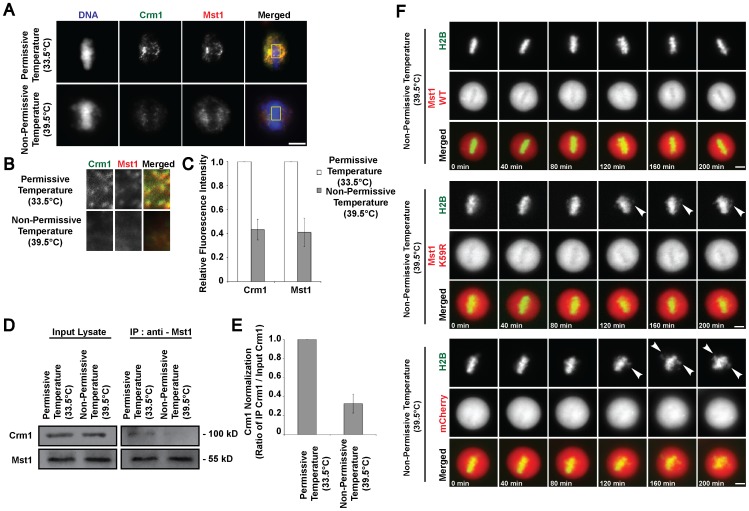
Crm1-Mst1-Aurora B kinase axis dictates the maintenance of stable kinetochore-microtubule attachments. A) Mitotic tsBN2 cells were immunostained with anti-Crm1 and anti-Mst1 following incubation at permissive or non-permissive temperature. B) Magnified images of the boxed regions illustrating anti-Crm1 and anti-Mst1 staining (magnified merged image is exclusive of DNA). C) Quantified Crm1 and Mst1 intensities were normalized and presented as relative fold change ± s.d. (error bar) of three independent experiments. D) Co-immunoprecipitation assay was conducted using monoclonal anti-Mst1 antibody on mitotic tsBN2 cell lysates harvested 4 hours after incubation at permissive or non-permissive temperature. E) Quantified Crm1 intensities were normalized and presented as relative fold change ± s.d. (error bar) of three independent experiments. F) Time-lapse imaging of metaphase tsBN2 cells expressing H2B-GFP and Mst1 WT-mCherry, Mst1 K59R-mCherry or mCherry (positive control). Overexpression of Mst1 WT abrogated the misalignment phenotype in cells incubated at non-permissive temperature. Arrows pointed to misaligned chromosomes. Scale bar: 10 µm.

In dissecting the molecular pathway in which RanGTP regulates stable kinetochore-microtubule attachments, we examined it from the perspective of its role in transport regulations. As such, we looked into RanGTP-Crm1-NES-bearing cargo ternary complex formation. Under normal physiological conditions, Crm1 localizes on the kinetochores of metaphase cells (Fig. S4A–B). However, when tsBN2 cells were incubated at non-permissive temperature, Crm1 was significantly reduced at the kinetochores. From various NES-bearing protein candidates tested, we found that Mst1 mimics Crm1’s localization in the absence and presence of mitotic RanGTP. Mst1 co-localizes with Crm1 on the spindle and at the kinetochores. As the temperature increased to non-permissive temperature, the absence of Crm1 at the kinetochores led to the loss of Mst1 at the site (Fig. 4B). To determine whether there is any difference in the physical interaction between Crm1 and Mst1 after temperature increase, we performed co-immunoprecipitation assay using anti-Mst1 antibody. Western blot analysis indicated that whilst Crm1 and Mst1 protein levels remained similar in the input lanes, there was considerably less Crm1 protein co-immunoprecipitated in the temperature-shifted samples (Fig. 4D–E). These results indicate that the depletion of RanGTP led to the delocalization of Crm1 and the subsequent failure to recruit Mst1 to the kinetochores.

To further validate the contribution of Mst1 in relation to RanGTP levels, we performed rescue experiments by overexpressing Mst1 WT or Mst1 kinase dead K59R mutant in tsBN2 cells and subsequently incubating the mitotic cells at non-permissive temperature. Time-lapse images showed that cells with Mst1 WT-mCherry overexpression reinstated properly aligned metaphase chromosomes even at non-permissive temperature (Fig. 4F, top panel). However, kinase-dead mutant, Mst1 K59R-mCherry transfection exhibited chromosome misalignment when RanGTP was abrogated (Fig. 4F, middle panel). Therefore, our data revealed that RanGTP-dependent recruitment of active Mst1 is necessary for the maintenance of stable kinetochore-microtubule attachments.

### Active Mst1 Interacts with and CURBS AURORA B Kinase Activity to Preserve Stable Kinetochore-microtubule Attachments

**Figure 5:**
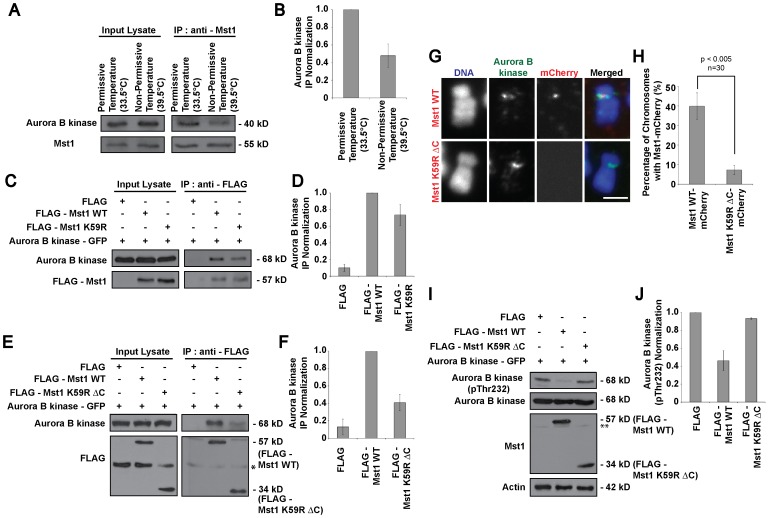
Robust interaction of functionally active Mst1 with Aurora B kinase for its inhibitory effect. A) Co-immunoprecipitation assay conducted using anti-Mst1 antibody on mitotic tsBN2 cell lysates harvested 4 hours after incubation at permissive or non-permissive temperature. B) Quantified Aurora B kinase intensities were normalized and presented as relative fold change ± s.d. (error bar) of three independent experiments. C) Immunoprecipitation assay conducted using monoclonal anti-FLAG antibody on metaphase-enriched HEK cell lysates co-transfected with plasmids as indicated. D) Quantified Aurora B kinase intensities were normalized against immunoprecipitated Aurora B kinase co-transfected with FLAG-Mst1 WT (middle lane) and presented as relative fold change ± s.d. (error bar) of three independent experiments. E) Immunoprecipitation assay conducted using monoclonal anti-FLAG antibody on metaphase-enriched HEK cell lysates co-transfected with plasmids as indicated. F) Quantified Aurora B kinase intensities were normalized against immunoprecipitated Aurora B kinase co-transfected with FLAG-Mst1 WT (middle lane) and presented as relative fold change ± s.d. (error bar) of three independent experiments. Asterisk (*) indicates non-specific bands. G) Metaphase spread of tsBN2 cells expressing Mst1 WT or K59R ΔC-mCherry were immunostained with anti-Aurora B kinase. Scale bar: 2 µm. Images were acquired with fixed-exposure mode. H) Histogram shows percentage of metaphase chromosomes with Mst1-mCherry fusion protein. Error bars show ± s.d. from three independent experiments. I) Western blot analysis of metaphase-enriched HEK cells co-transfected with Aurora B kinase and FLAG-Mst1 as indicated. Asterisk (**) indicates endogenous Mst1. Actin was used as loading control. J) Quantified Aurora B kinase (pThr232) intensities were normalized and presented as relative fold change ± s.d. (error bar) of three independent experiments.

Next, we proceeded to identify a downstream target of Mst1, which may be implicated in the presentation of the observed aberrant chromosome alignment phenotype. We found that endogenous Aurora B kinase was co-immunoprecipitated with Mst1 at permissive temperature, but there was significantly less Aurora B kinase co-immunoprecipitated from samples incubated at non-permissive temperature (Fig. 5A–B). This result indicates that Aurora B kinase may be a downstream substrate of Mst1 whose activity is affected by the loss of RanGTP. To further examine the involvement of Mst1 and Aurora B kinase along our proposed RanGTP-Crm1-NES-bearing cargo axis, additional co-immunoprecipitation assays were conducted using Mst1 mutants. Although pulldown with the Mst1 K59R mutant showed some reduction in Aurora B kinase binding, a more significant observation was that the Mst1 K59R ΔC (amino acid 1–330, lacking NES, and kinase activity) mutant associates only weakly with Aurora B kinase as compared to both the Mst1 WT and Mst1 K59R (Fig. 5C–F). Furthermore, immunofluorescence analysis on metaphase chromosome spreads showed that intact NES is necessary for the shuttling of Mst1 within the vicinity of the chromosome including the kinetochore thus facilitating its interaction with Aurora B kinase. Western blot analysis shows distinct overexpression of the Mst1-mCherry fusion proteins (Fig. S5B). Whilst Mst1 WT-mCherry fusion protein can be detected, Mst1-K59R ΔC-mCherry was clearly absent from the metaphasic chromosomal precinct (Fig. 5G–H). These results indicate that the presence of the NES on Mst1 is crucial and necessary for recruitment to the kinetochores via the RanGTP-Crm1 axis.

Interestingly, upon examination of the levels of active Aurora B kinase, we found that wild-type Mst1 might negatively regulate the autophosphorylation of Aurora B kinase. The levels of active Aurora B kinase were suppressed in the presence of overexpressed FLAG-Mst1 WT but remained high for samples with control FLAG plasmid or Mst1 K59R ΔC mutant plasmid transfection (Fig. 5I–J). This suggests that wild-type Mst1 negatively regulates the phosphorylation state of Aurora B kinase. The robust interaction between Mst1 and Aurora B kinase allows Mst1 to exert its inhibitory effect on Aurora B kinase. In other words, the NES-RanGTP dependant spatial regulation and functionally intact Mst1 are necessary for the binding of Mst1 with Aurora B kinase in order to regulate the phosphorylation states of Aurora B kinase. More importantly, our data suggest a link between Mst1, Aurora B kinase activity, and chromosomal misalignment upon RanGTP depletion.

### Hyperactivated Aurora B Kinase Activity in RanGTP-depleted Cells Leads to Failure to Maintain Stable Kinetochore-microtubule Attachments

**Figure 6:**
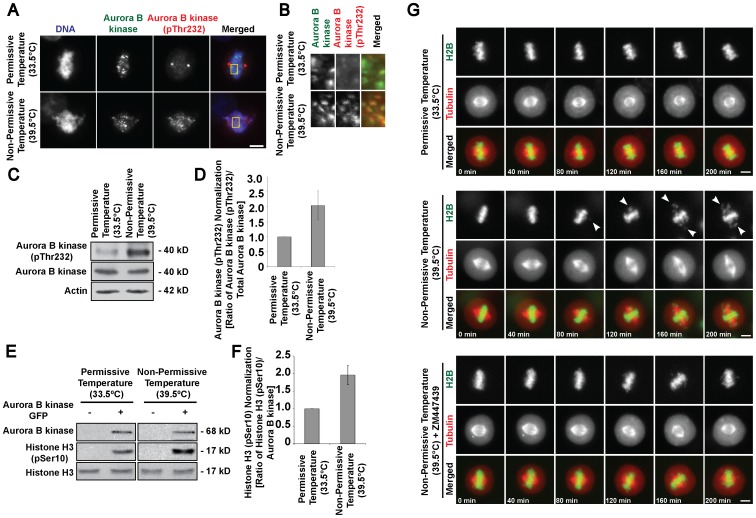
Aberrant Aurora B kinase activation upon RanGTP depletion leads to aberrant chromosomal alignment. A) Mitotic tsBN2 cells incubated at permissive or non-permissive temperature were analyzed by immunofluorescence staining with anti-Aurora B kinase and anti-Aurora B kinase (pThr232). Scale bar: 10 µm. B) Magnified images of the boxed regions illustrating anti-Aurora B kinase and anti-Aurora B kinase (pThr232) staining. Magnified merged image is exclusive of DNA. C) Western blot analysis of mitotic tsBN2 cells incubated at permissive or non-permissive temperature and harvested via mechanical shake-off. Actin was used as loading control. D) Quantified Aurora B kinase (pThr232) intensities were normalized and presented as relative fold change ± s.d. (error bar) of 3 independent experiments. E) Aurora B kinase assay was conducted using Aurora B kinase protein immunoprecipitated from mitotic tsBN2 cells incubated at permissive or non-permissive temperature. Kinase activity was determined by phosphorylation of a known Aurora B kinase substrate, Histone H3. F) Quantified histone H3 (pSer10) intensities were normalized and presented as relative fold change ± s.d. (error bar) of 3 independent experiments. G) Time-lapse imaging of metaphase tsBN2 cells expressing H2B-GFP and tubulin-mCherry. Control experiment (upper panel), temperature-shift experiment (middle panel), and temperature-shift + ZM447439 (bottom panel). Scale bar: 10 µm.

The activity and influence of Aurora B kinase on the dynamics of kinetochore-microtubule attachments have been shown to be dependent on the phosphorylation state of the kinase at Threonine 232 [22], [23]. Therefore, we examined the level of active Aurora B kinase (pThr232) at permissive and non-permissive temperature. Immunofluorescence staining for active phospho-Aurora B kinase showed both qualitative and quantitative enhanced fluorescence intensity for the temperature-shifted cells (Fig. 6A–B and Fig. S6A). Immunoblotting analysis of active Aurora B kinase from mitotic cells incubated at permissive or non-permissive temperature showed that although total amount of Aurora B kinase remains similar, there was a significantly higher level of active Aurora B kinase in RanGTP-depleted mitotic cells (Fig. 6C–D). To ascertain that Aurora B kinase activity is elevated in cells incubated at non-permissive temperature, an *in vitro* kinase assay on recombinant histone H3 was conducted with co-immunoprecipitated Aurora B kinase from mitotic cells incubated at permissive or non-permissive temperature. The kinase activity of Aurora B kinase was enhanced in the temperature-shifted sample, evident from an increase in histone H3 (pSer10) phosphorylation (Fig. 6E–F).

To further validate the influence of Aurora B kinase activity on the maintenance of metaphase chromosome alignment, we used a known Aurora B kinase inhibitor (ZM447439), which was added with MG132 for 2 hours before incubation at either permissive or non-permissive temperature. Time-lapse and immunofluorescence data (Fig. 6G and Fig. S6C) revealed that the misalignment phenotype was considerably suppressed when cells were treated with ZM447439. Quantification of the percentage of time-lapse imaged cells confirmed that there was significantly reduced percentage of metaphase cells with misaligned chromosomes when treated with ZM447439 (Fig. S6B). Interestingly, we noticed the occurrence of a population of metaphase cells with a milder degree of chromosome misalignment. For quantification analysis, we score for major misalignment as large apparent chromosome clusters grossly displaced from the metaphase plate whereas minor misalignment describes metaphase cells with less than 3 minuscule ‘lagging’ chromosome clusters. Normal chromosomal alignment was denoted by tightly packed aggregation of chromosomes at the equator of the cell. Quantification of more than 100 cells for each experimental set indicated that there was a significant reduction in the proportion of cells with major chromosome misalignment when temperature-shifted cells were treated with ZM447439 (Fig. S6D). Additionally, we show that the stability of spindle microtubules is preserved when ZM447439 is used to restrict the activity of Aurora B kinase at non-permissive temperature (Fig. S6E–F). Since active Aurora B kinase renders the kinetochore-microtubule attachment more labile, the stability of proper end-on attachments is greatly affected and thus leads to major chromosomal misalignment in the absence of RanGTP.

## Discussion

With the use of the Rango biosensor and FRET based on the correction-Youvan method, we have developed an approach that allows real-time visualization of the changes in RanGTP levels in parallel to the phenotypic alterations in tsBN2 cells. With minimal photobleaching effect, our new method enables continuous monitoring of chromosome orientation (or theoretically any experimental subjects of interest) relative to the fluctuations in RanGTP distribution at single cell level. Therefore, this method is not limited to observing processes that occur within a short duration. It is possible to follow a cell’s progression from interphase through the distinct phases of mitosis and to monitor cellular processes, which may be perturbed by changes in RanGTP levels as a cell progresses through the cell cycle.

Previous studies on *Xenopus* egg extracts and *C. elegans* embryos have shown that perturbation in RanGTP levels can result in aberrant chromosome alignment. However, these studies were conducted under conditions where Ran mutants were used to disrupt the RanGTP distribution as a cell enters mitosis or at prometaphase, prior to reaching metaphase and the observations are often accompanied by defective mitotic spindles [10], [24], [25]. To our knowledge, no study had reported a direct correlation between RanGTP and the maintenance of kinetochore-microtubule attachments at metaphase. In our experimental setup, cells were arrested at metaphase and metaphase chromosomes that have already achieved proper kinetochore-microtubule attachments should adopt a stable alignment of chromosomes at the metaphase plate. Intriguingly, our results indicate that upon RanGTP depletion, there was a progressive displacement of pre-aligned metaphase chromosomes from the equator, and thus suggesting an unprecedented regulatory role for RanGTP in modulating kinetochore-microtubule attachments at metaphase. With sufficient supporting evidence from the parallel experiments using control tsBN2 cells incubated at permissive temperature and the parental BHK21 cell line, we are able to demonstrate that the aberrant chromosome alignment phenotype is attributed to the loss of RCC1 and RanGTP depletion only.

Whilst we do note the concern regarding the use of a drug (MG132) to arrest cells at metaphase, this is necessary to trap the cells at metaphase to ensure that the depletion of RCC1 occurs during metaphase itself. The specific metaphase arrest would thus allow us to isolate the observed chromosome misalignment event and exclude any influence of other RanGTP-dependent mitotic processes prior to and after metaphase. This is especially important as the degradation of RCC1 and the subsequent depletion of RanGTP takes 2–3 hours (Fig. 2B). Additionally, the use of MG132 does not affect microtubule dynamics or RCC1 depletion at non-permissive temperature. Therefore MG132 is considered a suitable tool to arrest tsBN2 cells in this study. Although a similar chromosome misalignment/scattering phenotype was reported in a recent discovered phenomenon called ‘cohesion fatigue’ following prolonged SAC arrest [26], [27], we can rule out the likelihood of the occurrence of this phenomenon in our observed phenotype as we were able to observe intact sister chromatids with closely paired kinetochores from our 3D projection images as well as from chromosome spread images (Fig. S2B–D). Additionally, we were able to rescue the chromosome misalignment phenotype with wild-type RCC1 overexpression. This further affirms an involvement of mitotic RanGTP in maintaining proper chromosome alignment.

**Figure 7:**
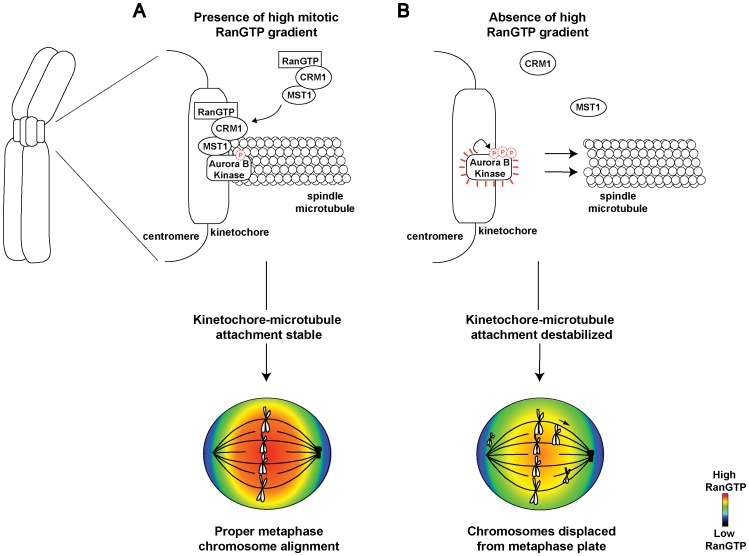
Model illustrating the role of mitotic RanGTP in sustaining stable chromosomal alignment during metaphase. In the presence of high mitotic RanGTP, Crm1 is localized to the kinetochores. Mst1 is then recruited as part of the RanGTP-Crm1-Mst1 ternary complex. The presence of Mst1 limits the autophosphorylation of Aurora B kinase and thus stabilizes kinetochore-microtubule attachments, which, results in stable chromosome alignment at the metaphase plate (A). When RanGTP is depleted, Crm1 is unable to bind and target Mst1 to the kinetochore. Subsequently, Aurora B kinase becomes hyperactivated to promote kinetochore-microtubule reorientation. The loss of proper amphitelic attachment ensues leading to the displacement of metaphase chromosomes from the equator (B).

Although previous studies on RanGTP have established its role in spindle formation [10], [28], [29], the involvement of RanGTP in the maintenance of kinetochore-microtubule attachments at metaphase has yet to be established. Our results describe a new role for RanGTP that bridges the molecular chronological gap between chromosome congression and chromosome segregation. We propose that the depletion of RanGTP during metaphase leads to the failure of Crm1 to recruit Mst1 to the kinetochores. Subsequently, the unrestricted hyperphosphorylation of Aurora B kinase promotes the destabilization of kinetochore-microtubule attachments and stimulation of promiscuous reorientation of kinetochore-microtubule attachments (Fig. 7). In agreement, Crm1 was significantly reduced at the kinetochores when mitotic RanGTP is depleted. Aberrantly aligned chromosomes were also detected when Leptomycin B was used to inhibit Crm1 function (Fig. S4D–F) [30].

Although Mst1 is an established cargo of Crm1 during interphase, its localization in mitosis is yet to be reported. Our finding here on the novel localization of Mst1 at the kinetochores along the RanGTP-Crm1-Mst1 axis is in accordance with the RanGTP-Crm1 dependent recruitment of NES proteins for kinetochore microtubule nucleation and subsequent chromosome segregation at anaphase [30], [31]. Additionally, co-immunoprecipitation and immunofluorescence results show that the localization and interaction of Mst1 with Aurora B kinase are dependent on the presence of a functional NES on Mst1. This is consistent with earlier reports highlighting the importance of the NES-bearing regulatory region on Mst1 for its localization and for regulation of its substrates such as FOXO1 and H2B [32].

To date, Aurora B kinase’s role at the kinetochore is established as a protagonist to rectify erroneous kinetochore-microtubule attachments rather than as the instigator of aberrant attachment and chromosome orientation [23], [33]. However, it is important to note that whilst the pre-established tension sensor and error correction role is indispensible during the search and capture process for kinetochore-microtubule attachment to form and stabilize, it is likely that after the formation of proper metaphase chromosome alignment, this role of Aurora B kinase becomes less prominent as it shifts towards regulating SAC signaling and anaphase onset [34], [35]. In the context of this study, proper chromosome alignment has already formed prior to RanGTP depletion and subsequent appearance of chromosomes displaced from the equator. Therefore it is likely that at this transitional stage between chromosome congression and segregation, the unrestricted hyperphosphorylation of Aurora B kinase due to the loss of RanGTP at metaphase inadvertently causes Aurora B kinase to promote promiscuous kinetochore-microtubule reorientation, resulting in the observed chromosome misalignment phenotype. Since the chromosome misalignment phenotype at non-permissive temperature can be rescued with the use of ZM447439 (Aurora B kinase inhibitor), and with Mst1 overexpression, it is likely that Aurora B kinase’s role at the kinetochore is negatively altered in the absence of RanGTP. Not surprisingly, upregulation of Aurora B kinase activity has been associated with genomic instability and oncogenic transformations in human cells. Considering Aurora B kinase’s role in establishing proper kinetochore-microtubule attachments and ensuring equal partitioning of genetic materials into daughter cells, precise regulation of Aurora B kinase activity is crucial to prevent the formation of aneuploid or euploid cells, which culminates towards cancer [36], [37]. Hence, our study here revealed RanGTP as the unifying positioning system that links the spatial regulation of Mst1-Aurora B kinase interaction to keep Aurora B kinase’s activity in check, thereby maintaining genomic integrity during cell division.

In conclusion, we have shown that the presence of the mitotic RanGTP is indeed critical for the maintenance of kinetochore-microtubule attachments. By promoting stable chromosome alignment and thus ensuring equal chromosome segregation, this regulatory role of RanGTP governs the precise process of chromosome partitioning.

## Materials and Methods

### Cell Culture and Drug Treatments

tsBN2 cells [38] and BHK21 cells were grown in DMEM (Gibco, Invitrogen, USA) containing 10% fetal bovine serum (Hyclone) and 1% Penicillin/Streptomycin (Gibco) in a humidified atmosphere with 5% carbon dioxide, at 33.5°C (permissive temperature). The non-permissive temperature used was 39.5°C. HEK cells were cultured in similar conditions but at 37°C. For MG132 treatment, 20 mM MG132 (Sigma, USA) in dimethyl sulfoxide (DMSO) stock was diluted in medium to a working concentration of 10 µM. Cells were treated with 10 µM of MG132 prior to incubation at 33.5°C or 39.5°C in experiments. Aurora B kinase inhibitor, ZM447439 (Tocris Biosciences, USA), was dissolved in DMSO to a stock concentration of 100 mM, and diluted in DMEM to a working concentration of 500 nM. Leptomycin B (Sigma) was dissolved in DMSO to a stock concentration of 5 µg/ml, and diluted in DMEM to a working concentration of 5 ng/ml. Transfection of Rango, histone H2B (H2B)-mCherry, H2B-GFP, tubulin-mCherry, wild-type (WT) RCC1-GFP, Aurora B kinase-GFP, Mst1 WT-mCherry, Mst1 K59R-mCherry, FLAG, FLAG-Mst1 WT, FLAG Mst1 K59R and/or FLAG-Mst1 K59R ΔC were performed using Lipofectamine 2000 (Invitrogen) according to manufacturer’s instructions. For cold-induced depolymerization assay, cells on coverslips were incubated in ice-cold DMEM at 4°C for 1 hour, then proceeded with immunofluorescence as described below.

### Immunofluorescence Microscopy

Cells seeded onto 22 mm coverslips were fixed with 100% ice-cold methanol at –20°C for 10 min. After washing thrice with TBST, cells were incubated with primary antibody diluted in TBST (50 mM Tris, pH 7.6, 150 mM NaCl, 0.1% Tween-20) plus 4% bovine serum albumin (BSA; Sigma, USA), at room temperature (RT) for 1.5 hour. Following washes with TBST, the cells were incubated with appropriate secondary antibodies and incubated at RT for 1 hour in the dark. Cells were mounted onto glass slides using ProLong Gold Antifade reagent containing DAPI (Invitrogen).

For immunofluorescence analysis of Crm1, cells were permeabilized with 40 µg/ml digitonin in transport buffer (20 mM HEPES, 110 mM potassium acetate, 5 mM sodium acetate, 2 mM magnesium acetate, 1 mM EGTA, 2 mM DTT) at 4°C for 6 min and fixed with 4% paraformaldehyde in phosphate buffered saline (PBS) for 15 min. After washing thrice with PBS, the cells were stained with antibodies and mounted on glass slides as described above.

For Mst1-mCherry chromosome staining, cells were incubated in hypotonic buffer (0.075 M KCl) at 37°C for 30 mins before centrifuging onto coverslips at 900 rpm for 4 mins in a cytocentrifuge (Cytospin 2, Thermo/Shandon). The cells were first permeabilized with 0.2% Triton X-100 in PBS at RT for 3 min and then fixed with 2% paraformaldehyde in PBS for 10 min. After two washes with PBS, cells were incubated with specific primary antibodies followed by fluorescent-labeled secondary antibodies and mounted onto glass slides.

Images were acquired and analyzed using an Axiovert 200 M inverted microscope (Carl Zeiss, Germany) and Axiovision 4.6 software.

### Chromosome Spread

Mitotic cells were collected by mechanical shake-off and incubated in hypotonic buffer at 37°C for 20 min. Following hypotonic swelling of the cells, the cells were pelleted by centrifugation at 2000 rpm for 5 min. After that, the cells were washed once and then fixed overnight with ice cold fixative solution (methanol : acetic acid at 3∶1) at 4°C. On the following day, the swollen fixed cells were dropped onto slides, dried and stained with ProLong gold Antifade reagent containing DAPI (Invitrogen). Chromosome spread images were acquired and analyzed using an Axiovert 200 M inverted microscope (Carl Zeiss, Germany) and Axiovision 4.6 software.

### Time-lapse Imaging

The effect of temperature increase to non-permissive temperature (39.5°C) on tsBN2 cells or transfected tsBN2 cells expressing Rango, H2B-mCherry, H2B-GFP, tubulin-mCherry, Mst1 WT-mCherry and/or Mst1 K59R-mCherry was followed by time-lapse microscopy. Cells were seeded onto 35 mm glass-bottom dishes or Ibidi 8-well chamber slides and placed on a heat-controlled stage of a Zeiss Axiovert 200 M microscope. The temperature was maintained at 33.5°C or increased to 39.5°C and CO_2_ levels were maintained at 5% using a CTI 3700 controller (Carl Zeiss). Phase contrast and fluorescent images were recorded (AxioCam camera and Axiovision 4.6 software) using a 40× objective at every 10 min interval. For time-lapse imaging of BHK21 cells, cells were transfected with tubulin-mCherry followed by time-lapse imaging using 20 × objective at every 10 min interval. For time lapse imaging of tsBN2 cells treated with Leptomycin B, cells were stained with Hoechst dye (Molecular Probes, USA) and imaged at 20 × objective at every 5 min interval.

### FRET and Image Analysis

With the use of the Rango biosensor, RanGTP levels were deduced via the fluorescence resonance energy transfer (FRET) method. Cells were maintained at 33.5°C or 39.5°C and 5% CO_2_ in a heat-controlled chamber on the microscope during FRET experiments. Physiological FRET images were recorded (AxioCam camera and Axiovision FRET 4.6 software) using a 40 × objective and FRET analysis was performed using the Axiovision FRET Correction-Youvan Method. The Correction-Youvan method measures the FRET value for an image and performs a ‘correction’ on the FRET value by deducting any crosstalk from the Donor and Acceptor.
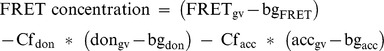
(gv = intensity as gray value, bg = background intensity, Cf = correction factor, FRET = FRET image, don = donor image, acc = acceptor image) [39].

### Spindle Microtubule Intensity (A.U.) and Relative Fluorescence Intensity Measurement

Images were acquired with the same exposure time using an Axiovert 200 M inverted microscope (Carl Zeiss) and analyzed with the Axiovision 4.6 software.
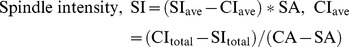



(CI = cell intensity, CA = cell area, SA = Spindle area).

Relative Aurora B kinase fluorescence intensity = pAurora Intensity_ave_/total Aurora B kinase Intensity_ave._

(Centrosome-specific stainings were excluded from measurements).

### Western Blotting

Mitotic cells were harvested by mechanical shakeoff, washed with PBS and lysed by 2 × sample buffer (62.5 mM Tris-HCl, pH 6.8, 2% SDS, 25% glycerol, 5% 2-mercaptoethanol, 0.01% bromophenol blue) containing 10% Complete EDTA-free Protease Inhibitor Cocktail (Roche, Switzerland) and 0.1% phosphatase cocktail inhibitor 1 and 2 (Sigma, USA). The mitotic lysate was boiled for 10 min. Equal amounts of protein were resolved on SDS-polyacrylamide gel and transferred onto nitrocellulose membranes (Biorad laboratories, USA). Membranes were probed with specific primary antibodies diluted in 10% skim milk in TBST at 4°C overnight. Membranes were washed thrice with TBST before incubating with horseradish peroxidase-conjugated anti-mouse IgG or anti-rabbit IgG or anti-goat IgG secondary antibodies (Invitrogen, USA). After washing with TBST, immunoreactive proteins were detected by enhanced chemiluminescence reagent (Amersham Biosciences, USA).

### Co-immunoprecipitation

Protein G-sepharose beads (Zymed), specified antibody and cell lysates were incubated at RT for 1 hour. The immunoprecipitates were washed with PBS thrice, and then 2 × sample buffer was added, boiled at 100°C to denature the proteins, separating them from the beads. Immunoprecipitated protein was analyzed by SDS-PAGE and western blotting.

### Immunoprecipitation for Kinase Assay

24 hours after transfection with Aurora B kinase-GFP, tsBN2 cells were treated with MG132 for 2 hours prior to incubation at permissive or non-permissive temperature for 4 hours. Then the cells were washed with PBS and lysed in buffer C (20 mM HEPES, pH 7.5, 10 mM KCl, 1.5 mM MgCl_2_ and 0.5% Nonidet P-40 subsitute) containing 10% Complete EDTA-free Protease Inhibitor Cocktail (Roche, Switzerland) and 0.1% phosphatase cocktail inhibitor 1 and 2 (Sigma). Lysates were incubated with protein G-sepharose beads (Zymed) and anti-Aurora B kinase mouse monoclonal antibody (BD Pharmigen) at RT for 1.5 hours. The immunoprecipitates were washed thrice with buffer C and once with buffer D (10 mM HEPES, pH 7.5, 10 mM KCl, 1 mM DTT and 10 mM MgCl_2_). Samples were resuspended in 20 µl of kinase mixture (20 mM HEPES, pH 7.5, 10 mM KCl, 1 mM DTT, 10 mM MgCl_2_ and 100 µM ATP). 6 µg of histone H3 (New England Biolabs) is added to each reaction mixture and reactions were incubated at 30°C for 30 min. The reactions were stopped by incubation on ice and the samples were eluted with 2± sample buffer and analyzed by SDS-PAGE and western blotting.

### Antibodies

The primary antibodies used were anti-actin goat polyclonal (Santa Cruz Biotechnology) used at 1∶3000, anti-RanGAP1 rabbit polyclonal, anti-Ran goat polyclonal (Santa Cruz Biotechnology), anti-securin mouse monoclonal, anti-GFP rabbit polyclonal (Abcam), anti-Crm1 mouse monoclonal (BD Pharmingen), anti-FLAG mouse monoclonal (Sigma), anti-Phospho-Aurora A (Thr288)/Aurora B (Thr232)/Aurora C (Thr198) and anti-Mst1 rabbit polyclonal antibody (Cell Signaling) used at 1∶1000 for immunoblotting, anti-RanGAP1 rabbit polyclonal, anti-TPX2 rabbit polyclonal, anti-Crm1 rabbit polyclonal (Santa Cruz Biotechnology), anti-BubR1 mouse monoclonal, anti-Crm1 mouse monoclonal, anti- Aurora B kinase/AIM mouse monoclonal, anti-Hec1 mouse monoclonal, anti-dynactin-p150 mouse monoclonal (BD Pharmingen) used at 1∶500, anti-human centromeric antigens (Antibodies Incorporated) used at 1∶10, anti-Mst1 rabbit polyclonal (Genetex, Incorporated) used at 1∶500, anti-Phospho-Aurora A (Thr288)/Aurora B (Thr232)/Aurora C (Thr198) (Cell Signaling) and anti-α-tubulin clone DM1α FITC-conjugate (Sigma) used at 1∶1000 for immunofluorescence. Secondary antibodies used for immunofluorescence were rhodamine Red-X-conjugated donkey anti-human IgG (Jackson Immunoresearch Laboratories Inc.) used at 1∶500, Alexa Fluor 594 goat anti-rabbit IgG, Alexa Fluor 488 goat anti-mouse IgG, Alexa Fluor 594 goat anti-mouse IgG (Invitrogen) used at 1∶1000.

## Supporting Information

Figure S1 **Mitotic spindle and metaphase chromosome alignment are unperturbed in parental BHK21 cell line at non-permissive temperature.** A) Time-lapse imaging of metaphase BHK21 cells (with endogenous wild-type RCC1 gene) transfected with tubulin-mCherry and stained with Hoechst dye (for DNA). B) Metaphase BHK21 cells immunostained with tubulin exhibiting intact mitotic spindle at both permissive and non-permissive temperature. Scale bar: 10 µm. C) Quantified histogram illustrating proportions of metaphase cells with intact mitotic spindle. (n = 30). Error bars show ± s.d. from three independent experiments.
(TIF)

Figure S2 **Ran regulators and sister chromatid cohesion are unperturbed by RanGTP disruption.** A) Western blot analysis of mitotic tsBN2 cells incubated at permissive or non-permissive temperature and harvested via mechanical shake-off at various time points. Actin was used as loading control. B) Chromosome spread images of mitotic tsBN2 cells incubated at permissive or non-permissive temperature. Magnified image of metaphase chromosome marked with asterisks (*) is shown inset, depicting tightly condensed metaphase chromosome with intact sister chromatids. Scale bar: 10 µm. C) Quantified histogram illustrating proportions of metaphase chromosomes with sister chromatids intact. (n = 30). Error bars show ± s.d. from three independent experiments. (Student’s t-test). D) 3D projection images of metaphase chromosomes of tsBN2 cell incubated at non-permissive temperature. Misaligned chromosomes possess intact sister chromatids with paired kinetochore staining (Hec1). E) Quantified histogram of the relative fluorescence intensity of RanGAP1 (grey) and BubR1 (white) for tsBN2 cells incubated at permissive or non-permissive temperature. (n = 30, 3 independent experiments). F) Representative images of metaphase tsBN2 cells incubated at permissive or non-permissive temperature and co-immunostained with BubR1 and RanGAP1. Scale bar: 10 µm.
(TIF)

Figure S3 **Localization of spindle protein TPX2, kinetochore proteins Hec1 and RanGAP1, and spindle motor dynactin-p150 are unaltered by RanGTP disruption.** Cells were immunostained with A) anti-TPX2 and tubulin B) anti-Hec1 and tubulin C) anti-RanGAP1 and tubulin D) anti-dynactin-p150 and tubulin. Scale bar: 10 µm.
(TIF)

Figure S4 **Crm1 is significantly reduced from the kinetochores after RanGTP disruption and inhibition of Crm1 with Leptomycin B results in misaligned chromosomes.** tsBN2 cells were immunostained with A) anti-Crm1 and anti-Hec1 B) anti-Crm1 and tubulin. Magnified images are shown on the right. Scale bar: 10 µm. C) Schematic depiction of the experimental conditions. tsBN2 cells were arrested at metaphase using MG132 for 2 hours, prior to treatment with Leptomycin B and imaged for time-lapse analysis or incubated for another 2 hours prior to fixing for immunofluorescence analysis. D) Quantified histogram of the proportion of fixed metaphase cells with misaligned chromosomes with or without Leptomycin B treatment at permissive temperature. (n = 30, 3 independent experiments). E) Representative images of metaphase cells with misaligned chromosomes with or without Leptomycin B treatment. Scale bar: 10 µm. F) Time-lapse imaging of metaphase tsBN2 cells stained with Hoechst dye (for DNA) and treated with Leptomycin B at permissive temperature. Arrows indicate aberrantly aligned chromosomes. Scale bar: 10 µm.
(TIF)

Figure S5 **NES-prevailing Mst1 is excluded from nucleus.** A) Interphase cells expressing Mst1 WT or K59R ΔC-mCherry were fixed and subjected to immunofluorescence analysis. Mst1 K59R ΔC mutant lacking NES shows distinct accumulation in the nucleus. Scale bar: 10 µm. B) Parallel western blot analysis of Mst1 and mCherry (DsRed) from mitotic tsBN2 cells transfected with Mst1 WT-mCherry or Mst1 K59R ΔC-mCherry (Fig. 5G) showing multifold increase in levels of Mst1 in cells transfected with Mst1-mCherry. C) Parallel western blot analysis of Mst1 and mCherry (DsRed) from mitotic tsBN2 cells transfected with mCherry control, Mst1 WT-mCherry or Mst1 K59R-mCherry as shown in Fig. 4F. Actin was used as loading control. D) Histogram shows percentage of time-lapse imaged metaphase cells with misaligned chromosomes from Fig. 4F (n = 25, 3 independent experiments). Error bars show ± s.d. from three independent experiments. (Student’s t-test).
(TIF)

Figure S6 **ZM447439 curbs the activity of Aurora B kinase to maintain the stability of kinetochore-microtubule attachments.** A) Histogram depicting quantification of relative Aurora B kinase (pThr232) fluorescence intensity normalized against total Aurora B kinase fluorescence intensity as mean ± s.d. from three independent experiment for cells imaged and presented in Fig. 6A and 6B. (n = 100). Images were acquired with fixed exposure time. (Student’s t-test). B) Histogram shows percentage of time-lapse imaged metaphase cells with misaligned chromosomes from Fig. 6G. Error bars show ± s.d. from three independent experiments. (Student’s t-test). C) Mitotic tsBN2 cells subjected to treatments as indicated. The cells were then fixed and stained with tubulin. Arrows indicate misaligned chromosomes. Scale bar: 10 µm. D) Quantified histogram illustrating proportion of metaphase cells with normal alignment, minor misalignment (<3 minuscule ‘lagging’ chromosome clusters) and major misalignment (>3 grossly displaced chromosomes clusters). E) Histogram shows total cold-stable microtubule intensity (A.U.) after cold-induced microtubule depolymerization treatments as indicated. Error bars show ± s.d. (Student’s t-test). F) Representative images of cold-treated mitotic spindles from mitotic tsBN2 cells incubated at permissive or non-permissive temperature. Scale bar: 10 µm.
(TIF)

## References

[1] Maiato H, DeLuca J, Salmon ED, Earnshaw WC (2004) The dynamic kinetochore-microtubule interface. Journal of cell science 117: 5461–5477. 15509863 10.1242/jcs.01536

[2] Cheeseman IM, Chappie JS, Wilson-Kubalek EM, Desai A (2006) The conserved KMN network constitutes the core microtubule-binding site of the kinetochore. Cell 127: 983–997. 17129783 10.1016/j.cell.2006.09.039

[3] DeLuca JG, Gall WE, Ciferri C, Cimini D, Musacchio A, et al (2006) Kinetochore microtubule dynamics and attachment stability are regulated by Hec1. Cell 127: 969–982. 17129782 10.1016/j.cell.2006.09.047

[4] Sundin LJ, Guimaraes GJ, Deluca JG (2011) The NDC80 complex proteins Nuf2 and Hec1 make distinct contributions to kinetochore-microtubule attachment in mitosis. Molecular biology of the cell 22: 759–768. 21270439 10.1091/mbc.E10-08-0671 PMC3057701

[5] McCleland ML, Kallio MJ, Barrett-Wilt GA, Kestner CA, Shabanowitz J, et al (2004) The vertebrate Ndc80 complex contains Spc24 and Spc25 homologs, which are required to establish and maintain kinetochore-microtubule attachment. Current biology: CB 14: 131–137. 14738735 10.1016/j.cub.2003.12.058

[6] Akiyoshi B, Sarangapani KK, Powers AF, Nelson CR, Reichow SL, et al (2010) Tension directly stabilizes reconstituted kinetochore-microtubule attachments. Nature 468: 576–579. 21107429 10.1038/nature09594 PMC3108429

[7] Gaitanos TN, Santamaria A, Jeyaprakash AA, Wang B, Conti E, et al (2009) Stable kinetochore-microtubule interactions depend on the Ska complex and its new component Ska3/C13Orf3. The EMBO journal 28: 1442–1452. 19360002 10.1038/emboj.2009.96 PMC2669960

[8] Kalab P, Pralle A, Isacoff EY, Heald R, Weis K (2006) Analysis of a RanGTP-regulated gradient in mitotic somatic cells. Nature 440: 697–701. 16572176 10.1038/nature04589

[9] Kalab P, Heald R (2008) The RanGTP gradient - a GPS for the mitotic spindle. Journal of cell science 121: 1577–1586. 18469014 10.1242/jcs.005959 PMC7185306

[10] Wilde A, Lizarraga SB, Zhang L, Wiese C, Gliksman NR, et al (2001) Ran stimulates spindle assembly by altering microtubule dynamics and the balance of motor activities. Nature cell biology 3: 221–227. 11231570 10.1038/35060000

[11] Ciciarello M, Mangiacasale R, Lavia P (2007) Spatial control of mitosis by the GTPase Ran. Cellular and molecular life sciences: CMLS 64: 1891–1914. 17483873 10.1007/s00018-007-6568-2 PMC11136129

[12] Clarke PR, Zhang C (2008) Spatial and temporal coordination of mitosis by Ran GTPase. Nature reviews Molecular cell biology 9: 464–477. 18478030 10.1038/nrm2410

[13] Kalab P, Weis K, Heald R (2002) Visualization of a Ran-GTP gradient in interphase and mitotic Xenopus egg extracts. Science 295: 2452–2456. 11923538 10.1126/science.1068798

[14] Li HY, Ng WP, Wong CH, Iglesias PA, Zheng Y (2007) Coordination of chromosome alignment and mitotic progression by the chromosome-based Ran signal. Cell cycle 6: 1886–1895. 17671426 10.4161/cc.6.15.4487

[15] Arnaoutov A, Dasso M (2003) The Ran GTPase regulates kinetochore function. Developmental cell 5: 99–111. 12852855 10.1016/s1534-5807(03)00194-1

[16] Nishitani H, Ohtsubo M, Yamashita K, Iida H, Pines J, et al (1991) Loss of RCC1, a nuclear DNA-binding protein, uncouples the completion of DNA replication from the activation of cdc2 protein kinase and mitosis. The EMBO journal 10: 1555–1564. 1851087 10.1002/j.1460-2075.1991.tb07675.x PMC452820

[17] Lampson MA, Renduchitala K, Khodjakov A, Kapoor TM (2004) Correcting improper chromosome-spindle attachments during cell division. Nature cell biology 6: 232–237. 14767480 10.1038/ncb1102

[18] Biggins S, Severin FF, Bhalla N, Sassoon I, Hyman AA, et al (1999) The conserved protein kinase Ipl1 regulates microtubule binding to kinetochores in budding yeast. Genes & development 13: 532–544. 10072382 10.1101/gad.13.5.532 PMC316509

[19] Brinkley BR, Cartwright J Jr (1975) Cold-labile and cold-stable microtubules in the mitotic spindle of mammalian cells. Annals of the New York Academy of Sciences 253: 428–439. 1056753 10.1111/j.1749-6632.1975.tb19218.x

[20] Musacchio A, Hardwick KG (2002) The spindle checkpoint: structural insights into dynamic signalling. Nat Rev Mol Cell Biol 3: 731–741. 12360190 10.1038/nrm929

[21] Kulukian A, Han JS, Cleveland DW (2009) Unattached kinetochores catalyze production of an anaphase inhibitor that requires a Mad2 template to prime Cdc20 for BubR1 binding. Dev Cell 16: 105–117. 19154722 10.1016/j.devcel.2008.11.005 PMC2655205

[22] Fuller BG, Lampson MA, Foley EA, Rosasco-Nitcher S, Le KV, et al (2008) Midzone activation of aurora B in anaphase produces an intracellular phosphorylation gradient. Nature 453: 1132–1136. 18463638 10.1038/nature06923 PMC2724008

[23] Santaguida S, Tighe A, D'Alise AM, Taylor SS, Musacchio A (2010) Dissecting the role of MPS1 in chromosome biorientation and the spindle checkpoint through the small molecule inhibitor reversine. The Journal of cell biology 190: 73–87. 20624901 10.1083/jcb.201001036 PMC2911657

[24] Silverman-Gavrila RV, Wilde A (2006) Ran is required before metaphase for spindle assembly and chromosome alignment and after metaphase for chromosome segregation and spindle midbody organization. Molecular biology of the cell 17: 2069–2080. 16481399 10.1091/mbc.E05-10-0991 PMC1415283

[25] Bamba C, Bobinnec Y, Fukuda M, Nishida E (2002) The GTPase Ran regulates chromosome positioning and nuclear envelope assembly in vivo. Current biology: CB 12: 503–507. 11909538 10.1016/s0960-9822(02)00741-8

[26] Stevens D, Gassmann R, Oegema K, Desai A (2011) Uncoordinated loss of chromatid cohesion is a common outcome of extended metaphase arrest. PloS one 6: e22969. 21829677 10.1371/journal.pone.0022969 PMC3149067

[27] Daum JR, Potapova TA, Sivakumar S, Daniel JJ, Flynn JN, et al (2011) Cohesion fatigue induces chromatid separation in cells delayed at metaphase. Current biology: CB 21: 1018–1024. 21658943 10.1016/j.cub.2011.05.032 PMC3119564

[28] Carazo-Salas RE, Gruss OJ, Mattaj IW, Karsenti E (2001) Ran-GTP coordinates regulation of microtubule nucleation and dynamics during mitotic-spindle assembly. Nature cell biology 3: 228–234. 11231571 10.1038/35060009

[29] Funabiki H, Murray AW (2000) The Xenopus chromokinesin Xkid is essential for metaphase chromosome alignment and must be degraded to allow anaphase chromosome movement. Cell 102: 411–424. 10966104 10.1016/s0092-8674(00)00047-7

[30] Arnaoutov A, Azuma Y, Ribbeck K, Joseph J, Boyarchuk Y, et al (2005) Crm1 is a mitotic effector of Ran-GTP in somatic cells. Nature cell biology 7: 626–632. 15908946 10.1038/ncb1263

[31] Torosantucci L, De Luca M, Guarguaglini G, Lavia P, Degrassi F (2008) Localized RanGTP accumulation promotes microtubule nucleation at kinetochores in somatic mammalian cells. Molecular biology of the cell 19: 1873–1882. 18287525 10.1091/mbc.E07-10-1050 PMC2366853

[32] Anand R, Kim AY, Brent M, Marmorstein R (2008) Biochemical analysis of MST1 kinase: elucidation of a C-terminal regulatory region. Biochemistry 47: 6719–6726. 18510339 10.1021/bi800309m PMC2844906

[33] Cimini D, Wan X, Hirel CB, Salmon ED (2006) Aurora kinase promotes turnover of kinetochore microtubules to reduce chromosome segregation errors. Current biology: CB 16: 1711–1718. 16950108 10.1016/j.cub.2006.07.022

[34] Santaguida S, Vernieri C, Villa F, Ciliberto A, Musacchio A (2011) Evidence that Aurora B is implicated in spindle checkpoint signalling independently of error correction. The EMBO journal 30: 1508–1519. 21407176 10.1038/emboj.2011.70 PMC3102279

[35] Maia AF, Feijao T, Vromans MJ, Sunkel CE, Lens SM (2010) Aurora B kinase cooperates with CENP-E to promote timely anaphase onset. Chromosoma 119: 405–413. 20354862 10.1007/s00412-010-0265-x

[36] Tatsuka M, Katayama H, Ota T, Tanaka T, Odashima S, et al (1998) Multinuclearity and increased ploidy caused by overexpression of the aurora- and Ipl1-like midbody-associated protein mitotic kinase in human cancer cells. Cancer research 58: 4811–4816. 9809983

[37] Ditchfield C, Johnson VL, Tighe A, Ellston R, Haworth C, et al (2003) Aurora B couples chromosome alignment with anaphase by targeting BubR1, Mad2, and Cenp-E to kinetochores. The Journal of cell biology 161: 267–280. 12719470 10.1083/jcb.200208091 PMC2172902

[38] Nishitani H, Kobayashi H, Ohtsubo M, Nishimoto T (1990) Cloning of Xenopus RCC1 cDNA, a homolog of the human RCC1 gene: complementation of tsBN2 mutation and identification of the product. Journal of biochemistry 107: 228–235. 2361953 10.1093/oxfordjournals.jbchem.a123031

[39] Malkusch W (2004) Quantitative Measurements Of FRET Using Standard Fluorescence Microscopy. Bioscience Technology. 32–34.

