# Supplementary figures and images for: In Vivo FRET Imaging Revealed a Regulatory Role of RanGTP in Kinetochore-Microtubule Attachments via Aurora B Kinase
Source: PLoS One. 2012 Sep 28;7(9):e45836. doi: 10.1371/journal.pone.0045836 (PMC3461030; doi:10.1371/journal.pone.0045836)

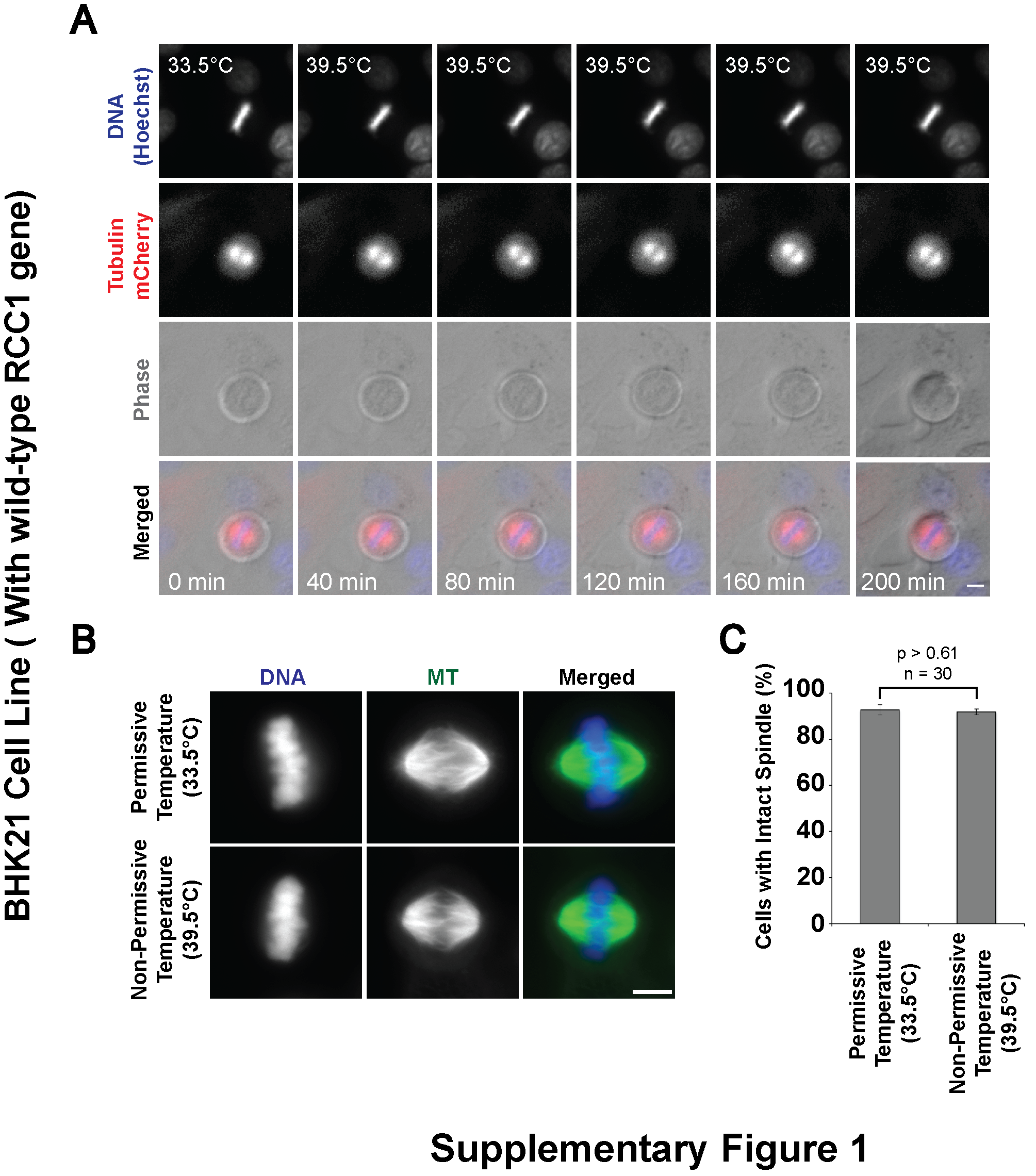

Supplement: Figure S1 — Mitotic spindle and metaphase chromosome alignment are unperturbed in parental BHK21 cell line at non-permissive temperature. A) Time-lapse imaging of metaphase BHK21 cells (with endogenous wild-type RCC1 gene) transfected with tubulin-mCherry and stained with Hoechst dye (for DNA). B) Metaphase BHK21 cells immunostained with tubulin exhibiting intact mitotic spindle at both permissive and non-permissive temperature. Scale bar: 10 µm. C) Quantified histogram illustrating proportions of metaphase cells with intact mitotic spindle. (n = 30). Error bars show ± s.d. from three independent experiments. (TIF) [file pone.0045836.s001.tif]

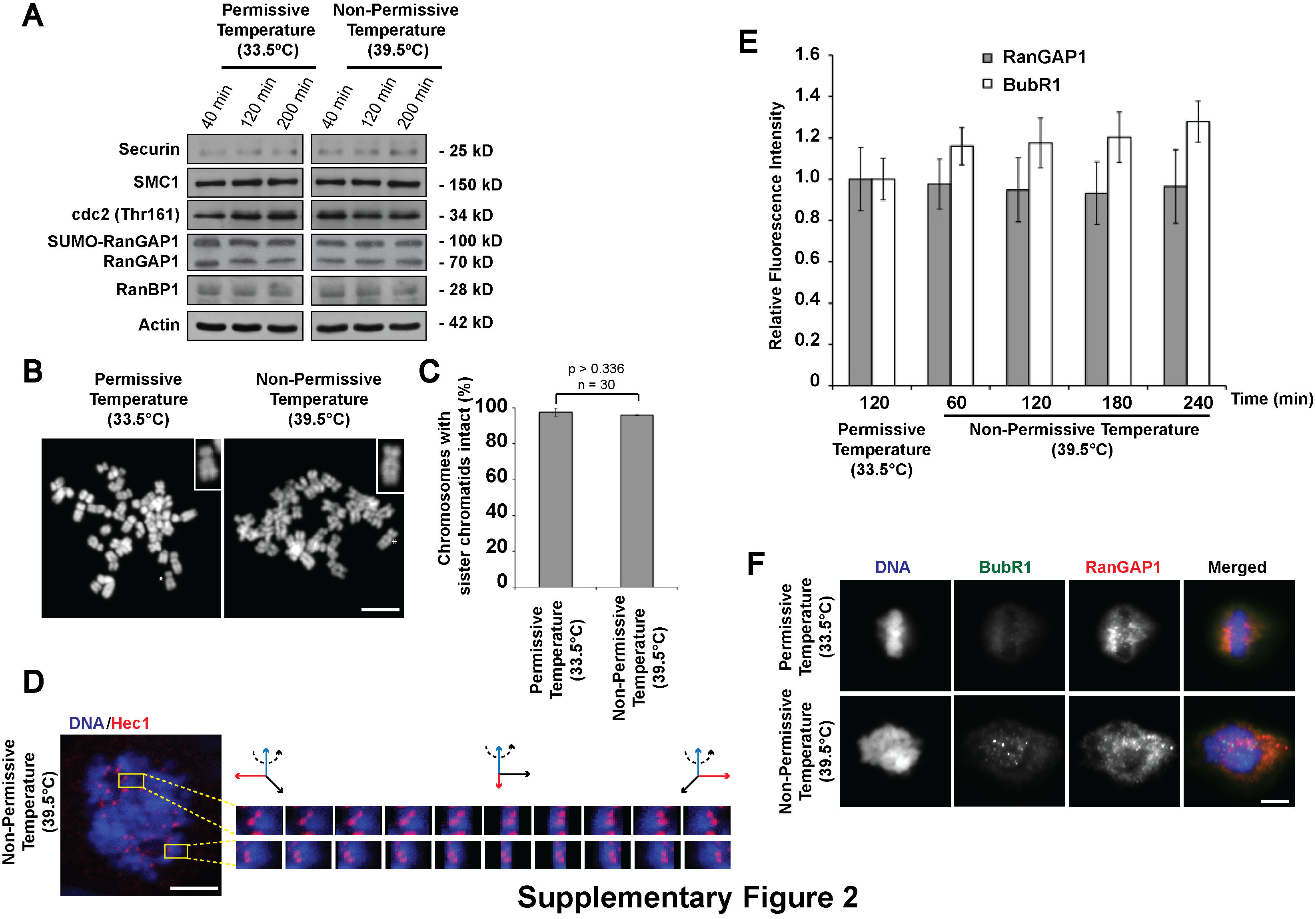

Supplement: Figure S2 — Ran regulators and sister chromatid cohesion are unperturbed by RanGTP disruption. A) Western blot analysis of mitotic tsBN2 cells incubated at permissive or non-permissive temperature and harvested via mechanical shake-off at various time points. Actin was used as loading control. B) Chromosome spread images of mitotic tsBN2 cells incubated at permissive or non-permissive temperature. Magnified image of metaphase chromosome marked with asterisks (*) is shown inset, depicting tightly condensed metaphase chromosome with intact sister chromatids. Scale bar: 10 µm. C) Quantified histogram illustrating proportions of metaphase chromosomes with sister chromatids intact. (n = 30). Error bars show ± s.d. from three independent experiments. (Student’s t-test). D) 3D projection images of metaphase chromosomes of tsBN2 cell incubated at non-permissive temperature. Misaligned chromosomes possess intact sister chromatids with paired kinetochore staining (Hec1). E) Quantified histogram of the relative fluorescence intensity of RanGAP1 (grey) and BubR1 (white) for tsBN2 cells incubated at permissive or non-permissive temperature. (n = 30, 3 independent experiments). F) Representative images of metaphase tsBN2 cells incubated at permissive or non-permissive temperature and co-immunostained with BubR1 and RanGAP1. Scale bar: 10 µm. (TIF) [file pone.0045836.s002.tif]

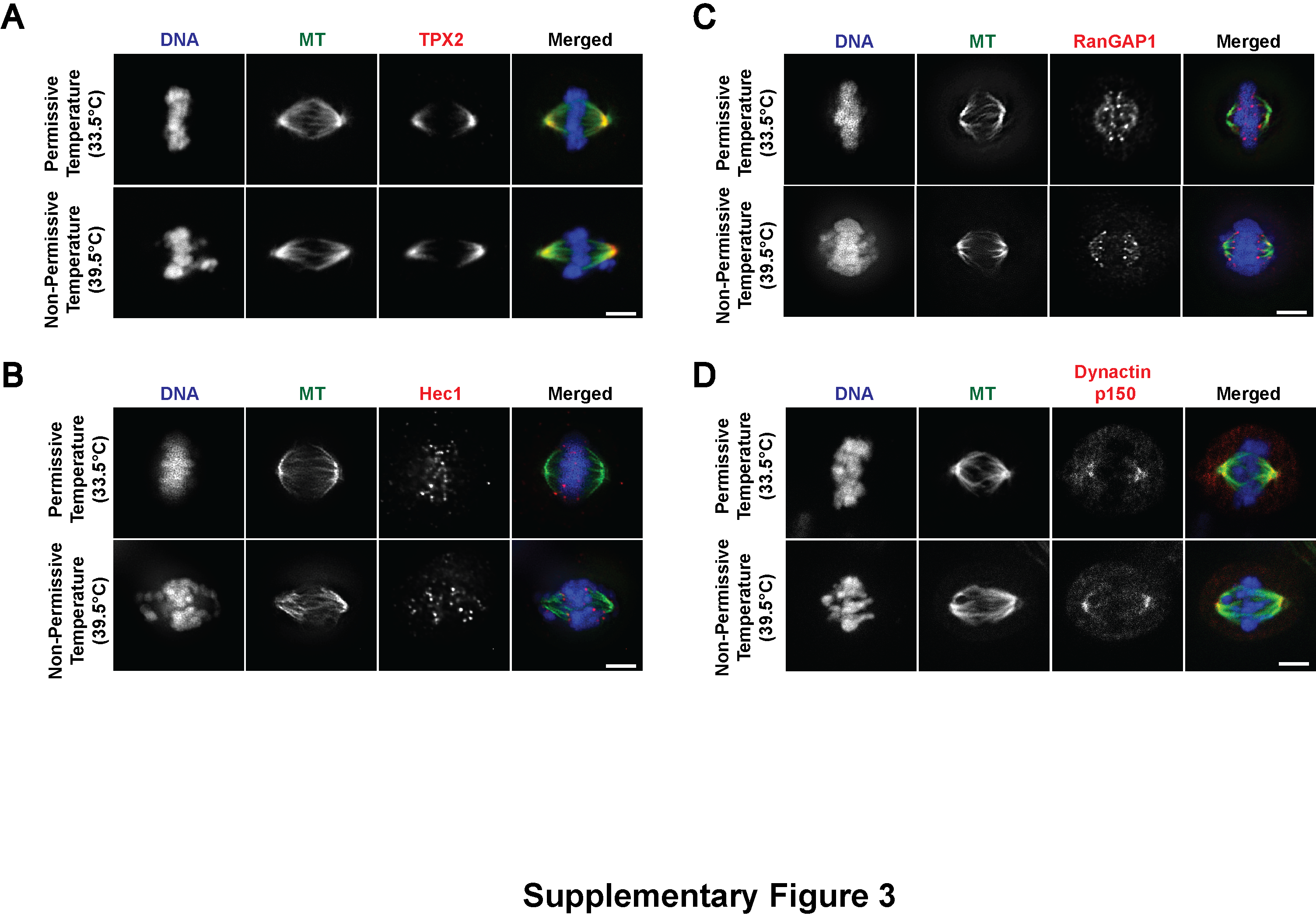

Supplement: Figure S3 — Localization of spindle protein TPX2, kinetochore proteins Hec1 and RanGAP1, and spindle motor dynactin-p150 are unaltered by RanGTP disruption. Cells were immunostained with A) anti-TPX2 and tubulin B) anti-Hec1 and tubulin C) anti-RanGAP1 and tubulin D) anti-dynactin-p150 and tubulin. Scale bar: 10 µm. (TIF) [file pone.0045836.s003.tif]

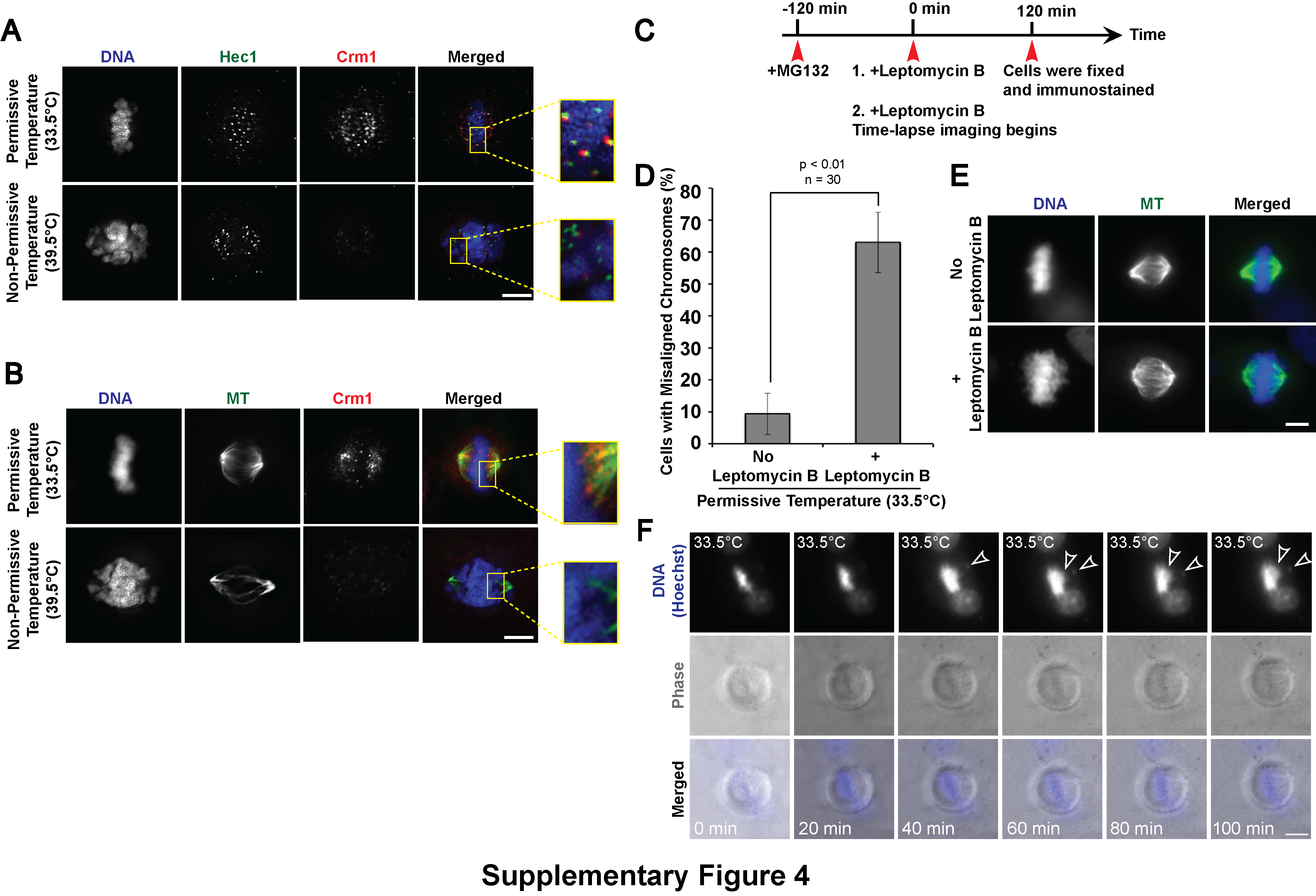

Supplement: Figure S4 — Crm1 is significantly reduced from the kinetochores after RanGTP disruption and inhibition of Crm1 with Leptomycin B results in misaligned chromosomes. tsBN2 cells were immunostained with A) anti-Crm1 and anti-Hec1 B) anti-Crm1 and tubulin. Magnified images are shown on the right. Scale bar: 10 µm. C) Schematic depiction of the experimental conditions. tsBN2 cells were arrested at metaphase using MG132 for 2 hours, prior to treatment with Leptomycin B and imaged for time-lapse analysis or incubated for another 2 hours prior to fixing for immunofluorescence analysis. D) Quantified histogram of the proportion of fixed metaphase cells with misaligned chromosomes with or without Leptomycin B treatment at permissive temperature. (n = 30, 3 independent experiments). E) Representative images of metaphase cells with misaligned chromosomes with or without Leptomycin B treatment. Scale bar: 10 µm. F) Time-lapse imaging of metaphase tsBN2 cells stained with Hoechst dye (for DNA) and treated with Leptomycin B at permissive temperature. Arrows indicate aberrantly aligned chromosomes. Scale bar: 10 µm. (TIF) [file pone.0045836.s004.tif]

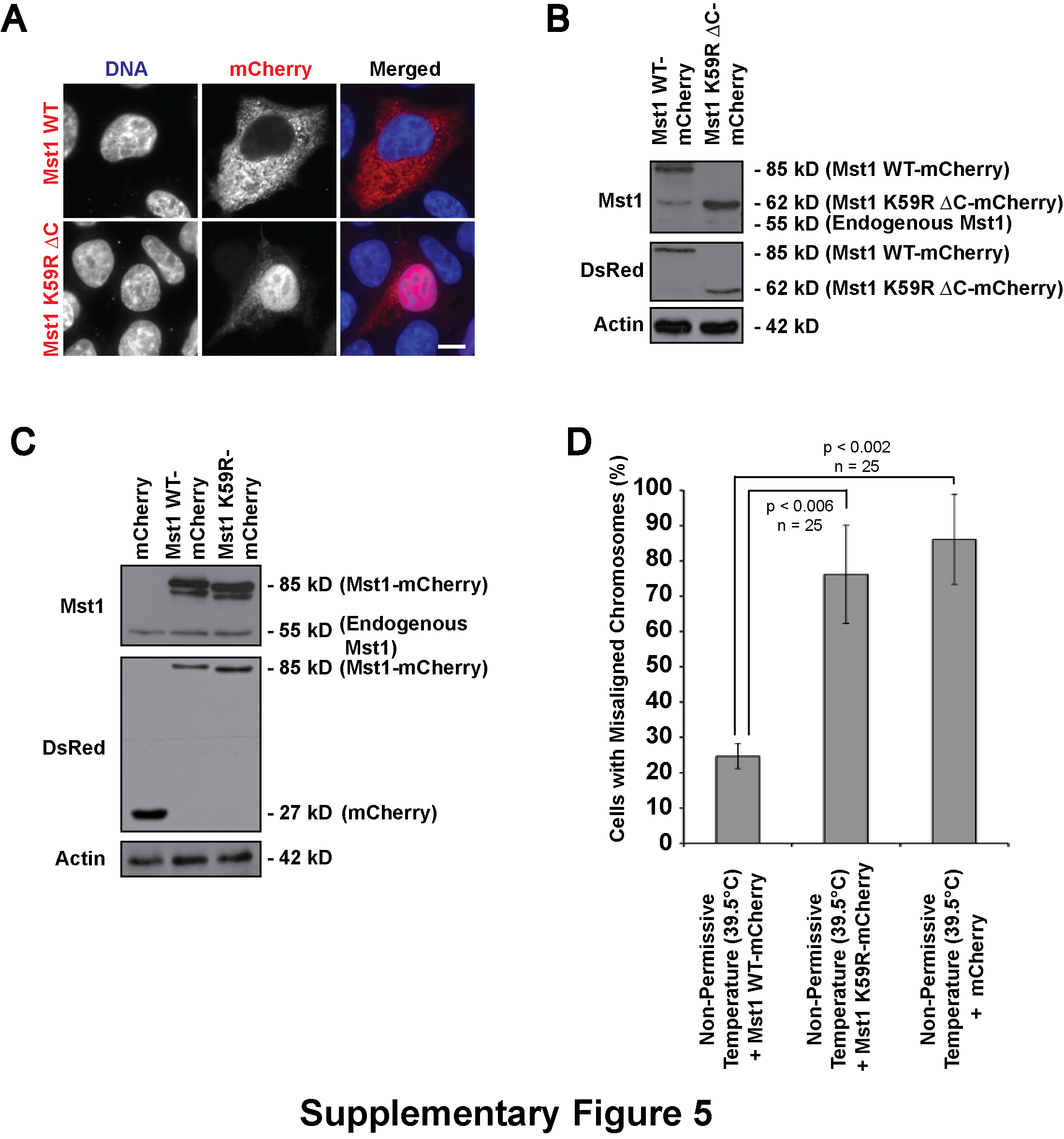

Supplement: Figure S5 — NES-prevailing Mst1 is excluded from nucleus. A) Interphase cells expressing Mst1 WT or K59R ΔC-mCherry were fixed and subjected to immunofluorescence analysis. Mst1 K59R ΔC mutant lacking NES shows distinct accumulation in the nucleus. Scale bar: 10 µm. B) Parallel western blot analysis of Mst1 and mCherry (DsRed) from mitotic tsBN2 cells transfected with Mst1 WT-mCherry or Mst1 K59R ΔC-mCherry (Fig. 5G) showing multifold increase in levels of Mst1 in cells transfected with Mst1-mCherry. C) Parallel western blot analysis of Mst1 and mCherry (DsRed) from mitotic tsBN2 cells transfected with mCherry control, Mst1 WT-mCherry or Mst1 K59R-mCherry as shown in Fig. 4F. Actin was used as loading control. D) Histogram shows percentage of time-lapse imaged metaphase cells with misaligned chromosomes from Fig. 4F (n = 25, 3 independent experiments). Error bars show ± s.d. from three independent experiments. (Student’s t-test). (TIF) [file pone.0045836.s005.tif]

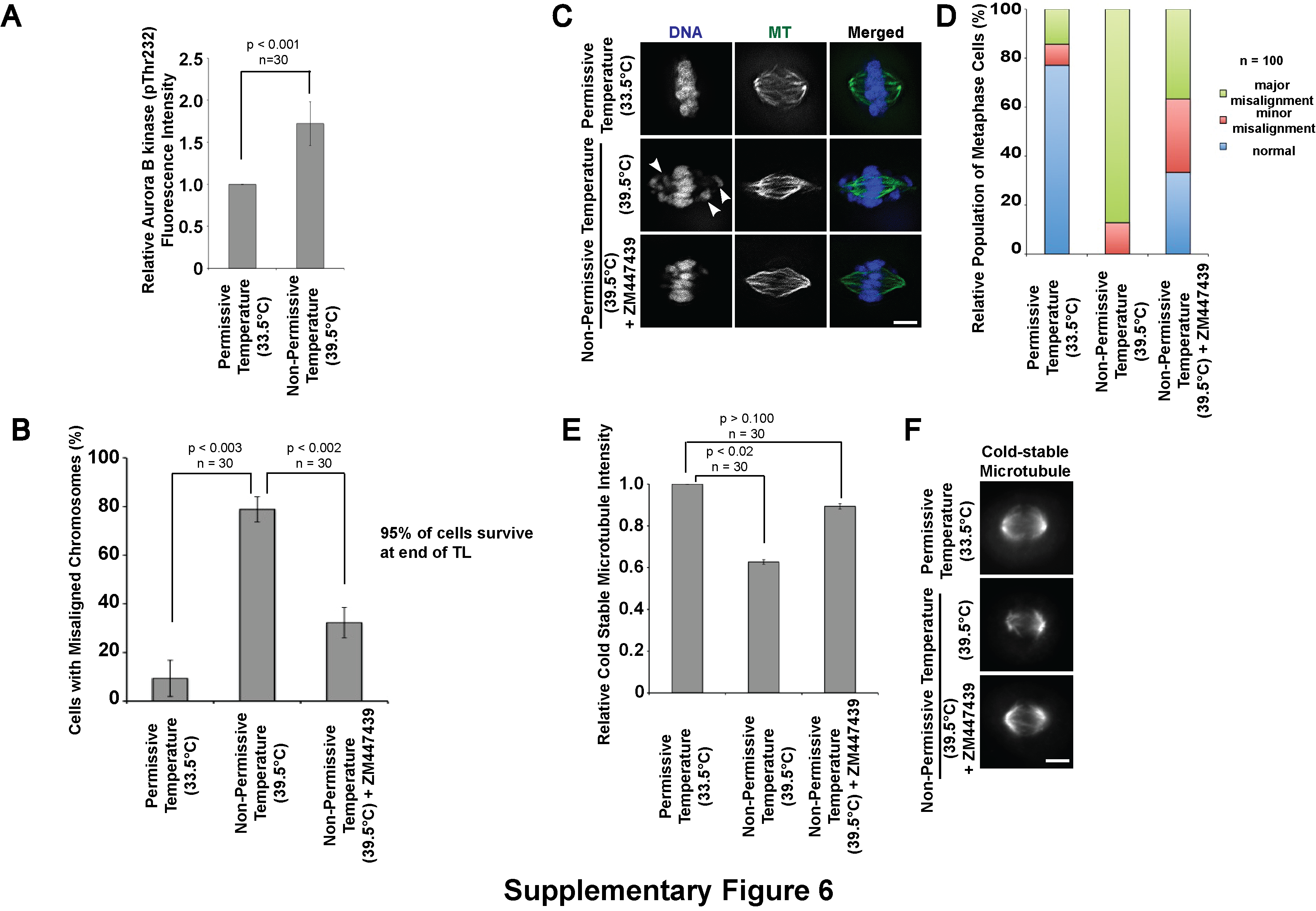

Supplement: Figure S6 — ZM447439 curbs the activity of Aurora B kinase to maintain the stability of kinetochore-microtubule attachments. A) Histogram depicting quantification of relative Aurora B kinase (pThr232) fluorescence intensity normalized against total Aurora B kinase fluorescence intensity as mean ± s.d. from three independent experiment for cells imaged and presented in Fig. 6A and 6B. (n = 100). Images were acquired with fixed exposure time. (Student’s t-test). B) Histogram shows percentage of time-lapse imaged metaphase cells with misaligned chromosomes from Fig. 6G. Error bars show ± s.d. from three independent experiments. (Student’s t-test). C) Mitotic tsBN2 cells subjected to treatments as indicated. The cells were then fixed and stained with tubulin. Arrows indicate misaligned chromosomes. Scale bar: 10 µm. D) Quantified histogram illustrating proportion of metaphase cells with normal alignment, minor misalignment (<3 minuscule ‘lagging’ chromosome clusters) and major misalignment (>3 grossly displaced chromosomes clusters). E) Histogram shows total cold-stable microtubule intensity (A.U.) after cold-induced microtubule depolymerization treatments as indicated. Error bars show ± s.d. (Student’s t-test). F) Representative images of cold-treated mitotic spindles from mitotic tsBN2 cells incubated at permissive or non-permissive temperature. Scale bar: 10 µm. (TIF) [file pone.0045836.s006.tif]
